# Biodegradable Hydrogels Based on Chitosan and Pectin for Cisplatin Delivery

**DOI:** 10.3390/gels9040342

**Published:** 2023-04-17

**Authors:** Regina R. Vildanova, Svetlana F. Petrova, Sergey V. Kolesov, Vitaliy V. Khutoryanskiy

**Affiliations:** 1 Ufa Institute of Chemistry, Ufa Federal Research Centre of the Russian Academy of Sciences, 450054 Ufa, Russia; 2 School of Pharmacy, University of Reading, P.O. Box 224, Reading RG6 6AD, UK

**Keywords:** chitosan, pectin, hydrogel, drug delivery, cisplatin

## Abstract

Preparation of stable hydrogels using physically (electrostatically) interacting charge-complementary polyelectrolyte chains seems to be more attractive from a practical point of view than the use of organic crosslinking agents. In this work natural polyelectrolytes—chitosan and pectin—were used, due to their biocompatibility and biodegradability. The biodegradability of hydrogels is confirmed by experiments with hyaluronidase as an enzyme. It has been shown that the use of pectins with different molecular weights makes it possible to prepare hydrogels with different rheological characteristics and swelling kinetics. These polyelectrolyte hydrogels loaded with cytostatic cisplatin as a model drug provide an opportunity for its prolonged release, which is important for therapy. The drug release is regulated to a certain extent by the choice of hydrogel composition. The developed systems can potentially improve the effects of cancer treatment due to the prolonged release of cytostatic cisplatin.

## 1. Introduction

Today, hydrogels based on synthetic polymers are widely used as drug delivery systems [1,2,3]. At the same time, hydrogels based on natural polymers have a greater potential, since they have structural similarity to the tissues of living organisms, better biocompatibility, and low toxicity [4,5,6]. In contrast to chemical crosslinking, physical crosslinking leads to the production of hydrogels under mild conditions without the need to use toxic reagents and thus by absence a subsequent purification process [7,8]. Hydrogel is a system consisting of a crosslinked hydrophilic polymer network and a solvent. This solvent may also include some other molecules, such as a drug. While in chemically crosslinked hydrogels the polymer network is formed by covalent bonds, in physically crosslinked hydrogels the role of crosslinks is played by physical interactions such as hydrogen bonds, ionic bonds, or hydrophobic effects. The simplest way to prepare a physically crosslinked hydrogel is to mix solutions of oppositely charged polyelectrolytes, resulting in the formation of a polyelectrolyte complex that can exist in the form of a precipitate, nano- or microsized particles, or, under certain conditions, in the form of a hydrogel [9,10].

Chitosan is a polymer prepared by alkaline deacetylation of chitin, the main component of the outer shell of arthropods (cuticle of insects, shell of crustaceans), as well as most fungi and some algae [11,12]. The disadvantage of chitosan to a certain extent is its insolubility at pH > 6.5 [13], which can be overcome by using its water-soluble salts, such as hydrochloride. The polycationic nature of chitosan ensures its interaction with natural or synthetic polyanions. Pectins are anionic polysaccharides that are isolated by extraction from plant raw, fruits, vegetables, and mushrooms [14,15]. Several studies [16,17,18] describe physically crosslinked hydrogels based on chitosan and pectin; however, they differ in the conditions for preparing hydrogels and their properties. In our work, we describe for the first time the production of hydrogels based on chitosan and pectin for the delivery of cisplatin. Cisplatin is an inorganic substance, divalent platinum chloride–ammonia complex, cis-[Pt(NH_3_)_2_Cl_2_]. This drug has alkylating, immunosuppressive, antitumor, cytostatic properties and is used in the treatment of various tumors [19]. Topical administration of a hydrogel, containing cisplatin, can reduce the severity of side effects of this cytotoxic drug, such as neurotoxicity, ototoxicity, myelosuppression, nausea, and vomiting [20,21,22]. In addition, the slow prolonged release of the drug from the polymer matrix allows the dose of the drug to be increased, which makes it possible to reduce the frequency of drug administration and has a positive effect on the treatment.

In this work, the behavior of hydrogels based on chitosan hydrochloride and various pectins (extracted from apples and citrus fruits), with different rheological properties, was studied. The swelling of hydrogels differs depending on the content of polymers and the composition of the dispersion medium. The pattern of the degradation of the polymer matrix also depends on the characteristics of both the polymer carrier itself and the composition of the medium. The release of cisplatin from hydrogels was studied using high-performance liquid chromatography.

## 2. Results and Discussion

### 2.1. Preparation of Hydrogel Based on Chitosan and Pectin

**Figure 1:**
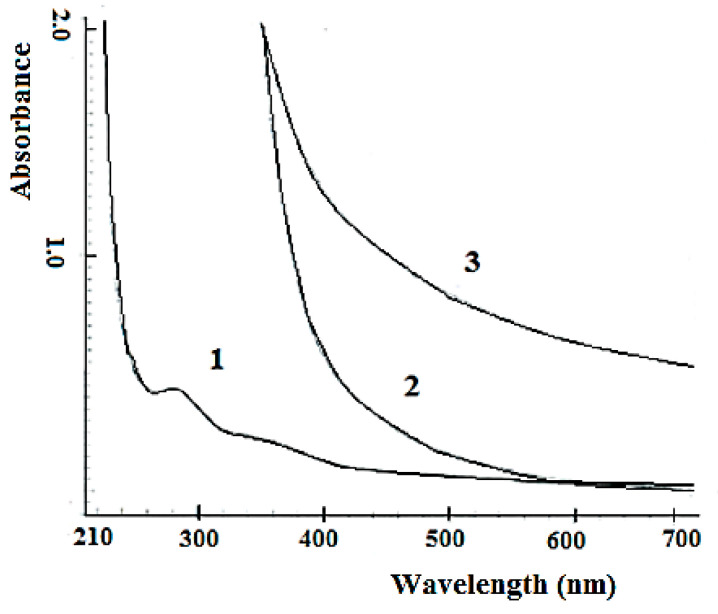
UV–Vis absorption spectra of pectin solution 0.5% (1), chitosan hydrochloride solution 2.0% (2), mixed solution of pectin and chitosan hydrochloride at the molar ratio 1:1 (3).

First, the interaction of dilute solutions of chitosan hydrochloride and pectin at an equimolar ratio of polymers was studied. When the solutions are mixed, a substantial increase in the viscosity of the system is observed, which is accompanied by an increase in the solution turbidity measured at 400–700 nm, however, hydrogel formation is not observed under these conditions (Figure 1). An increase in the concentration of polymers to 1% in the mixture leads to the formation of a polyelectrolyte complex in the form of a hydrogel, which is detected by the loss of fluidity of the system.

**Figure 2:**
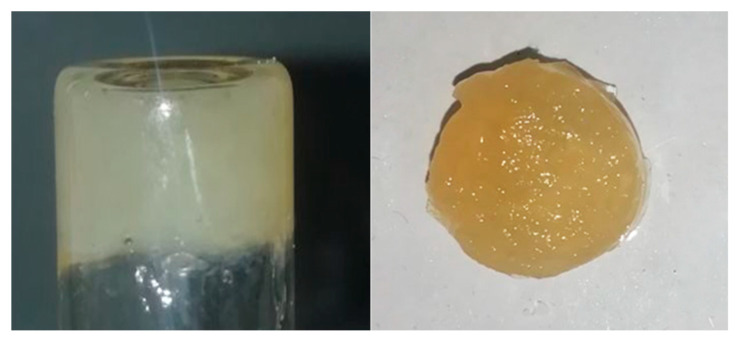
Physically crosslinked hydrogels formed in the equimolar mixtures (1:1) of chitosan hydrochloride and pectin.

When mixtures of chitosan and pectin were prepared in a saline solution, the vial should be shaken rapidly at room temperature during which a hazy pale-yellow hydrogel subsequently forms within a few seconds (Figure 2). When equivalent quantities of polymers were used, 100% yield of hydrogel without separation of the dispersion medium is reached. When changing the molar ratio of polymers, the hydrogel yield decreased. With an increase in the content of chitosan, the density of the crosslinked network increases, and a gradual syneresis of the dispersion medium and precipitation are observed.

**Scheme 1:**
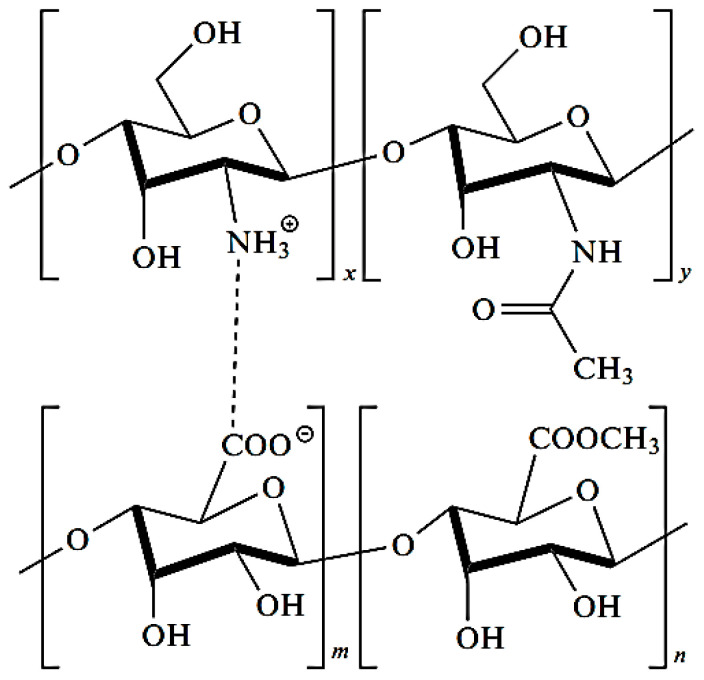
Scheme of electrostatic interactions between chitosan and pectin.

**Figure 3:**
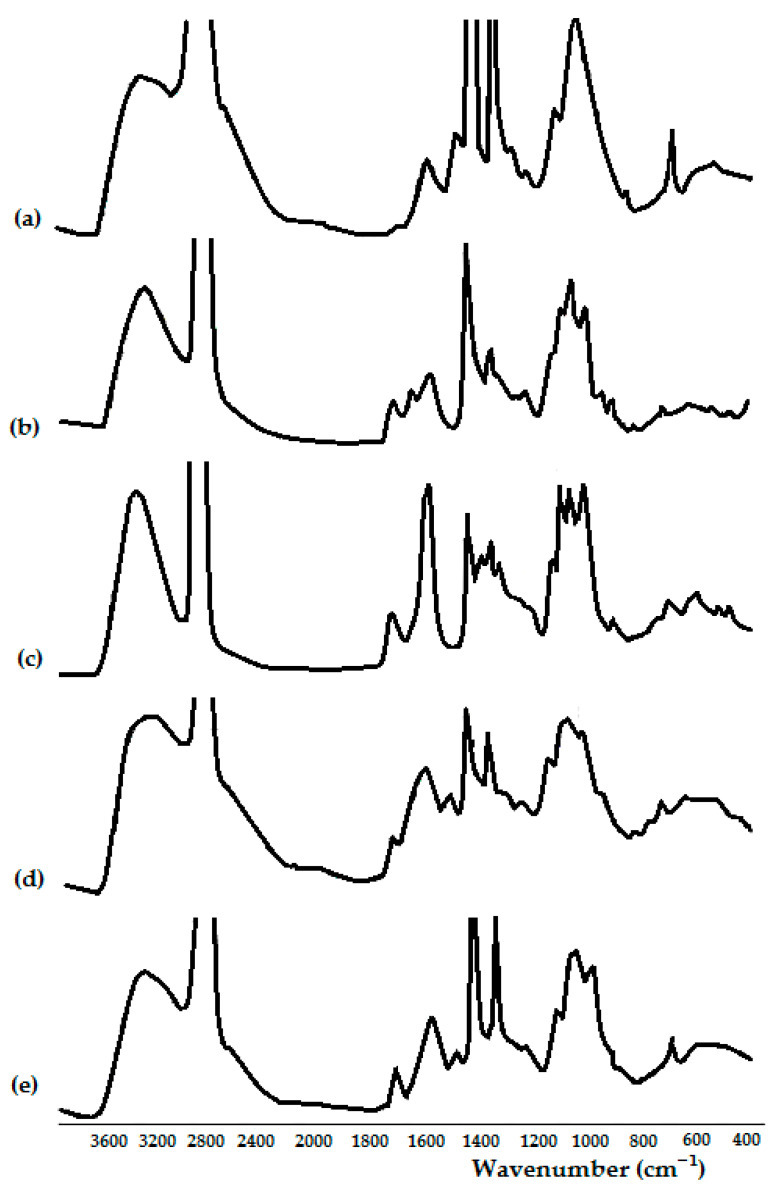
FT–IR spectra of (**a**) chitosan hydrochloride, (**b**) apple pectin, (**c**) citrus pectin, (**d**) chitosan hydrochloride/apple pectin 1:1 hydrogel, (**e**) chitosan hydrochloride/citrus pectin 1:1 hydrogel.

The formation of a physically crosslinked hydrogel is due to the electrostatic attractive interactions of the ammonium groups of chitosan and the carboxylate groups of pectin, as well as formation of many hydrogen bonds due to the interaction of a large number of hydrophilic groups of both polysaccharides, resulting in the formation of a polyelectrolyte complex (Scheme 1). The structure of the polyelectrolyte complex was confirmed by FT-IR spectroscopy data, recorded after drying the samples. Changes in the range of 1550–1800 cm^−1^ are observed in the spectrum of the polyelectrolyte complex, which are due to the interaction of ammonium groups of chitosan and carboxylate groups of pectin (Figure 3).

### 2.2. Rheological Properties of Polymer Solutions and Hydrogels

Formation of a physically crosslinked network in this system affects its structural and mechanical properties. These properties determine the possibility of practical use of these hydrogels for various purposes, therefore their further study was carried out.

**Figure 4:**
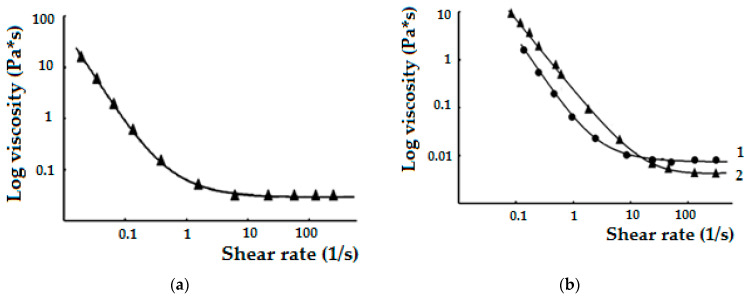
Viscosity as a function of shear rate for polymers, dissolved in saline solution: (**a**) chitosan hydrochloride (conc. 8%) or (**b**) pectin (conc. 2%): 1—apple; 2—citrus. Data are expressed as mean ± SD; *n* = 3.

Figure 4 shows the changes in viscosity of the initial polymers solutions with a change in the shear rate. As the velocity gradient increases, the structure of the polymer solution is disturbed and, accordingly, the viscosity decreases. The region of the highest Newtonian viscosity is absent, which is the case for undiluted solutions of rigid-chain polymers. The values of effective viscosities of citrus pectin solution are higher compared to apple pectin due to its higher molecular mass. Upon reaching a shear rate of 10 s^−1^, the viscosity of the polymer solutions decreases to the limit value and remains constant, which corresponds to the value of the lowest Newtonian viscosity. Thus, solutions of polyelectrolytes, chitosan hydrochloride and pectins, arenon-Newtonian liquids.

**Figure 5:**
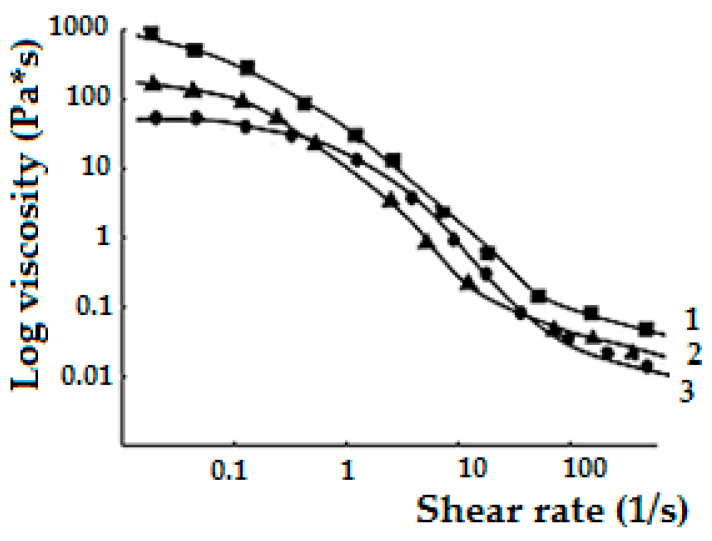
Viscosity as a function of shear rate for hydrogels: 1—chitosan hydrochloride/citrus pectin, T = 20 °C; 2—chitosan hydrochloride/apple pectin, T = 20 °C; 3—chitosan hydrochloride/apple pectin, T = 40 °C. Data are expressed as mean ± SD; *n* = 3.

Mixing solutions of chitosan hydrochloride and pectin leads to a significant increase in the viscosity of the system (Figure 5). At low shear rates, the viscosity of the chitosan hydrochloride/citrus pectin hydrogel is about four times higher than that of the chitosan hydrochloride/apple pectin hydrogel. As the temperature increases from 20 to 40 °C (curves 2 and 3), the viscosity decreases.

At *τ* < *τ*_0_, the hydrogel exhibits the properties of an elastic solid body; at *τ* > *τ*_0_, plastic flow occurs. The yield strength corresponding to the beginning of its plastic flow can be considered as a quantitative characteristic of the strength of the three-dimensional crosslinked structure of the hydrogel.

For hydrogels, a yield strength appears on the flow curves: chitosan hydrochloride/apple pectin hydrogel—4.1 ± 0.1 Pa, chitosan hydrochloride/citrus pectin hydrogel—12.2 ± 0.2 Pa, which is consistent with the molecular weights of pectins. Thus, based on the study of the rheological properties of hydrogels, it was shown that their flow obeys the Bingham law, i.e., these systems are plastic.

### 2.3. Swelling Kinetics of Hydrogels

**Figure 6:**
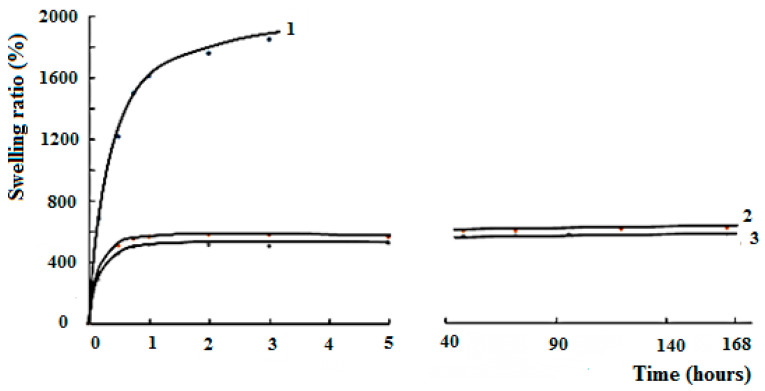
Swelling kinetics of chitosan hydrochloride/apple pectin hydrogels in distilled water (1); PBS (pH = 7.4) (2); saline solution (3). Data are expressed as mean ± SD; *n* = 3.

The gel formed based on chitosan hydrochloride and pectin was dried to a constant mass and was subsequently immersed in aqueous solutions and the change in the sample mass was monitored as a result of liquid sorption. As can be seen from Figure 6, the presence of inorganic ions in the composition of the hydrogel (curves 2, 3) leads to the swelling kinetics reaching a plateau within 1 h. At the same time, the value of the swelling ratio practically does not change within 4 days, which is manifested in the fact that under these conditions, sufficiently strong structures are formed that do not appear to degrade on long time.

**Figure 7:**
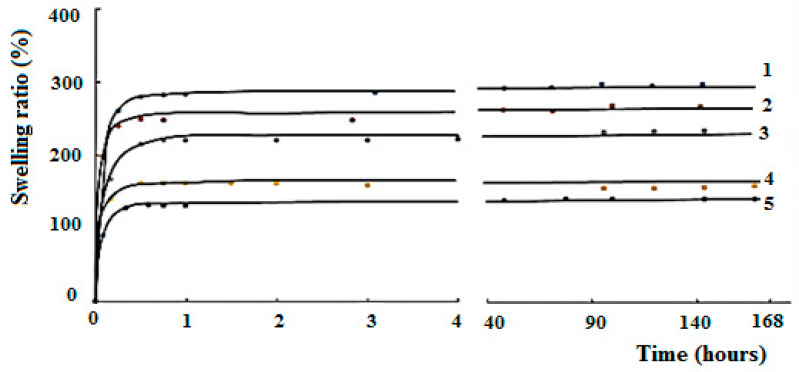
Swelling kinetics of chitosan hydrochloride/citrus pectin hydrogels in distilled water. Chitosan hydrochloride conc. 8% (1, 2) or 4% (3–5), citrus pectin conc. 2% (1, 3, 4) or 1% (2, 5), molar ratio chitosan/pectin 1:1 (1–3, 5) or 1:2 (4). Data are expressed as mean ± SD; *n* = 3.

Figure 7 shows that the gel prepared with the lowest total concentration of polysaccharides and their equimolar ratio is characterized by the lowest degree of swelling (curve 5). With a two-fold increase of the total polymer concentration (curve 1), the degree of swelling increases too. This phenomenon is apparently because the formation of hydrogels occurs from polymer solutions with obviously different viscosities and, accordingly, with different supramolecular organization. In the more dilute solution of polymer (4% chitosan hydrochloride or 1% pectin), by the moment of loss of fluidity, the emerging macromolecular structure has time to approach the equilibrium of close packing of chains to the greatest extent. The structure of a polymer body from a more concentrated solution (8% chitosan hydrochloride or 2% pectin) undergoes structural relaxation to a lesser extent. Accordingly, a looser packing of macrochains contributes to an increase in the degree of swelling.

**Figure 8:**
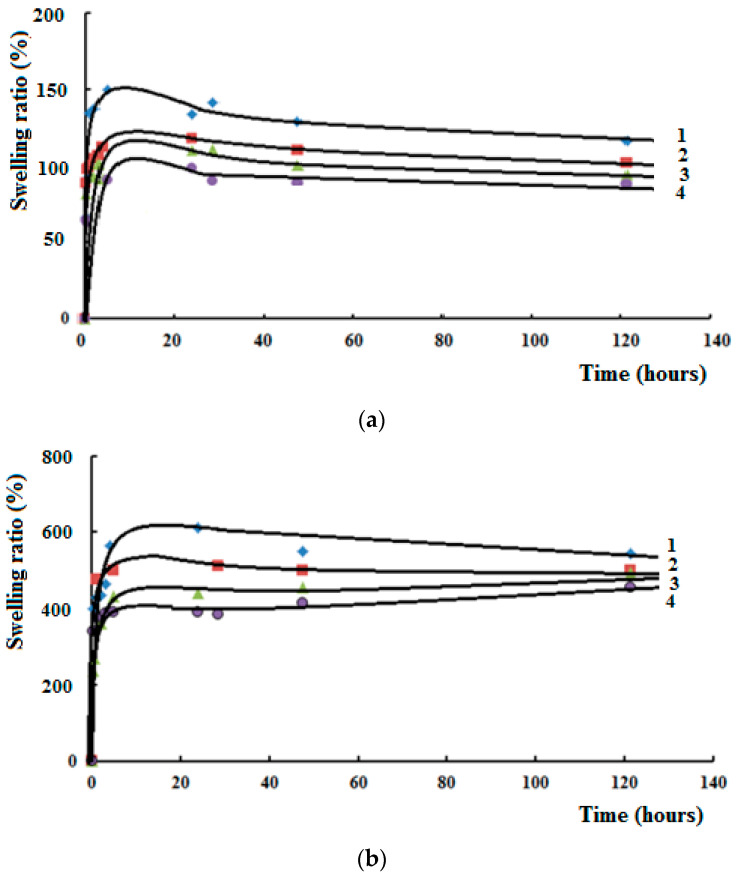
Swelling kinetics of hydrogels on the base of hydrochloride chitosan and citrus (**a**) or apple (**b**) pectin in distilled water (1), NaCl solution of different concentrations: 0.5 % (2), 0.9% (3), 2.0% (4). Data are expressed as mean ± SD; *n* = 3.

As follows from the data presented in Figure 8, the degree of swelling of the gel decreases with an increase in the concentration of sodium chloride in water, i.e., with an increase in the ionic strength of the solution. This results from the fact that the equilibrium swelling of polyelectrolyte complex hydrogels is determined by the balance of two forces, one of which is due to the elasticity of the network, and the second being due to the osmotic pressure caused by the counterions of the diffuse layer surrounding the fixed charges of the functional groups [23]. First, during swelling, almost one-sided diffusion of solvent molecules into the spatial network of the gel formed by polyelectrolyte complex takes place, i.e., osmosis of the solvent through the pores of a semipermeable membrane. Both processes, the release of ions and the diffusion of the solvent, are due to the tendency of the system to equalize the concentrations of the components. The swelling mechanism is reduced to the penetration of solvent molecules into the nearest polymer layers and solvation of the corresponding sections of polymer chains. As a result, there is a change in the conformations (unfolding) of segments of macromolecules, which facilitates penetration of solvent molecules further both into the gel (and an increase in its mass and volume) and out of the gel—the process of “deswelling” of the gel. When a dry sample is immersed in water, the osmotic pressure increases due to counterions inside the gel, and the maximum increase in the gel volume is thermodynamically beneficial. As a result, when the environment is deionized water, i.e., in the absence of electrolytes, the gel begins to rapidly increase its volume.

It is also worth noting that the nature of pectin affects the swelling process: gels based on apple pectin swell more, which is apparently due to the larger mesh size of the crosslinked network, compared to the gels based on citrus pectin. It is known that the mesh size is related to the rheological properties of hydrogels, the greater the hydrogel yield strength, the smaller the mesh size [24,25].

**Figure 9:**
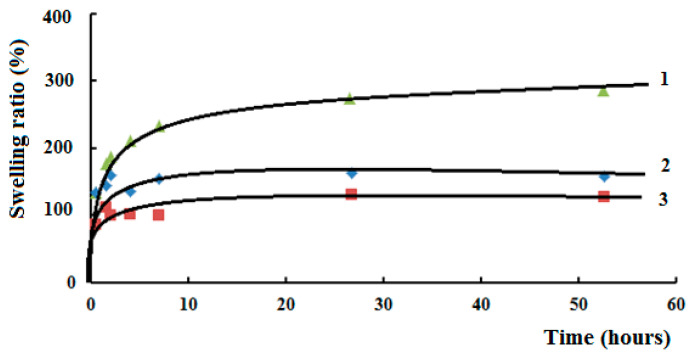
Swelling kinetics of chitosan hydrochloride/citrus pectin hydrogels in 0.9% solution of AlCl_3_ (1), CaCl_2_ (2), NaCl (3). Data are expressed as mean ± SD; *n* = 3.

A change in the nature of the inorganic salt (NaCl, CaCl_2_, AlCl_3_) in the swelling medium also leads to a change in the degree of swelling (Figure 9), what deals with pH of the environment. In the case of AlCl_3_ solution, it is acidic, and in an acidic medium, as follows from the data in Section 2.4. “In Vitro Stability”, in a time comparable to the time of the swelling experiment, the decomposition of the hydrogel proceeds by 10–15%. Accordingly, the network density decreases and the degree of swelling increases.

### 2.4. In Vitro Stability

When the hydrogel sample is kept in a saline solution over time, a mixture of polymers is released into the solution at a low rate. The degradation of the hydrogel is accompanied by the transition of the least hydrogen-bonded fibers from the surface of the polymer matrix into the dispersion medium, followed by their swelling and dissolution.

**Figure 10:**
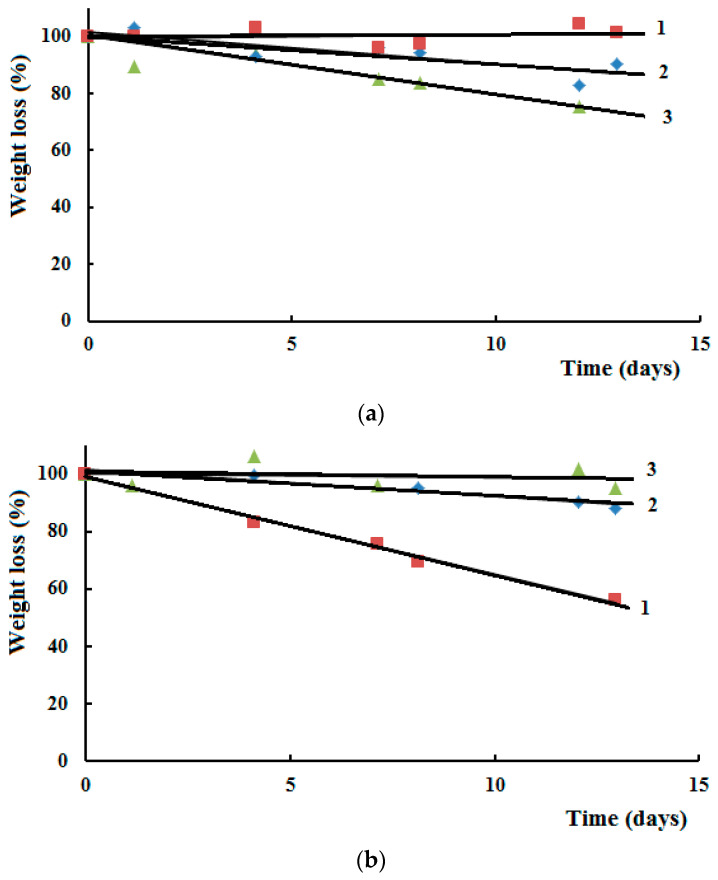
Weight loss of hydrogels with time in various dispersion media: saline solution (**a**) or PBS (**b**), immersed in saline solution (1), distilled water (2), PBS (3). Data are expressed as mean ± SD; *n* = 3.

The influence of the nature of the dispersion medium (saline solution or PBS) of the hydrogel on its degradation was studied (Figure 10). In both cases, there is a slight degradation of about 10% hydrogel within 2 weeks in distilled water. There is no degradation if the composition of the dispersion medium of the hydrogel coincides with the composition of the external environment; otherwise, this process is accelerated.

**Figure 11:**
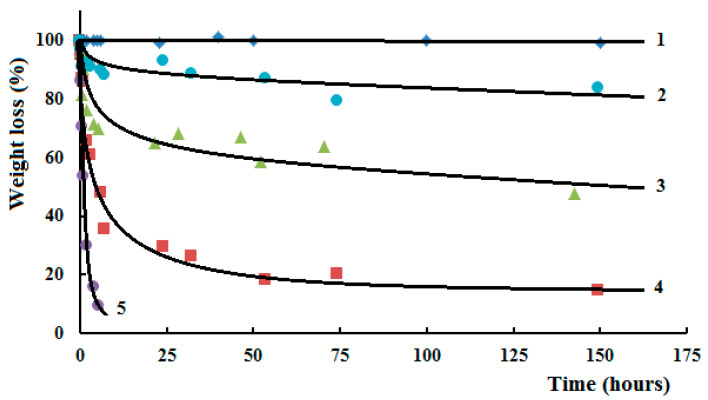
Weight loss of hydrochloride/apple pectin hydrogels: 1—distilled water; sodium phosphate buffers with different pH: 2—pH = 6.4; 3—pH = 5.8; 4—pH = 7.4; 5—pH = 8.0. Data are expressed as mean ± SD; *n* = 3.

The study of the pH effect on the degradation process showed that hydrogels quickly dissolve in an alkaline medium (pH = 8) within a few hours (Figure 11). At pH 5.8, the hydrogel degrades by about 40% in 6 days, while at pH 6.4, it degrades by 20% over the same time.

### 2.5. Enzymatic Degradation

Since the use of the developed hydrogels involves their contact with the living tissues in a human body with exposure to enzymes of various natures, an experiment was conducted to identify the effect of the hyaluronidase enzyme on the hydrogel and its degradation. Hyaluronidase is a specific enzyme that breaks hyaluronic acid down. At the same time, it was shown [26,27] that this enzyme successfully hydrolyzes chitosan in acidic solutions on the surface of films and reduces the particle size of polyelectrolyte complexes. Herewith, several researchers reported the inhibitory effect of pectin on hyaluronidase [28,29,30].

**Figure 12:**
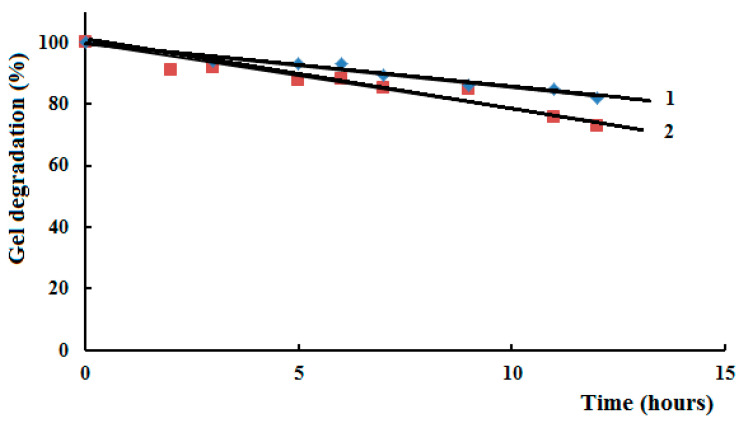
Hydrogel degradation by hyaluronidase 64 U/mL in saline solution at T = 20 °C (1) or 40 °C (2). Data are expressed as mean ± SD; *n* = 3.

As can be seen from Figure 12, the introduction of hyaluronidase at a concentration of 64 U/mL into the saline solution of the external environment leads to a gradual decrease in the mass of the hydrogel to a greater extent at a higher temperature.

### 2.6. In Vitro Retention of Cisplatin from Hydrogels

To prepare drug-containing hydrogels, a solution of cisplatin in saline was used as a solvent for polymers. The addition of cisplatin to hydrogels has practically no effect on their properties; at the same time, there is a slight decrease in the gelation time, probably due to additional physical interactions between the polymers and the drug. Thus, it was shown in [31] that the amino groups of cisplatin interact with the carboxyl groups of pectin.

**Figure 13:**
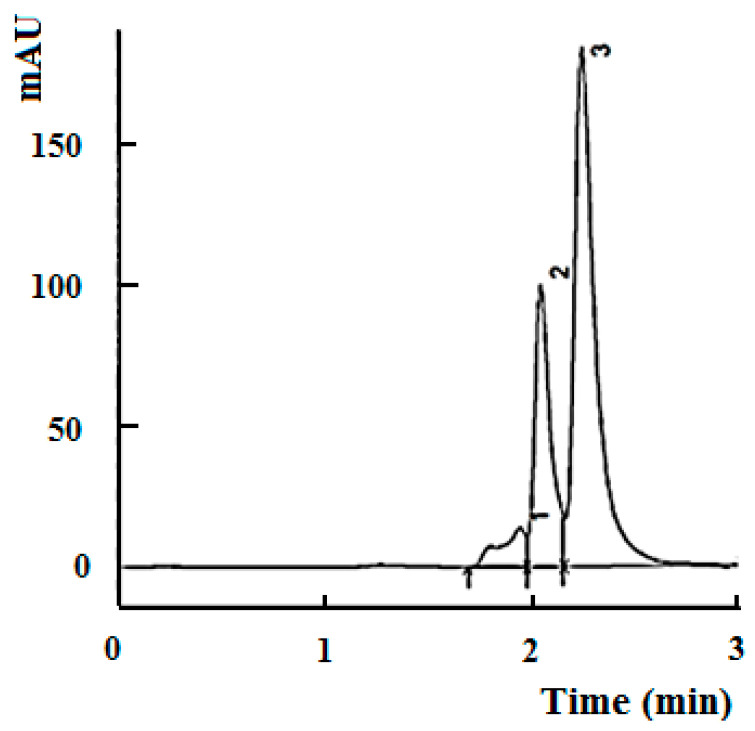
Chromatograph of solution after 45 min hydrogel immersion in saline solution, peaks: hydrochloride chitosan (1), apple pectin (2), cisplatin (3).

The release of cisplatin from the hydrogels was evaluated using high-performance liquid chromatography. A chromatogram of a cisplatin solution at a concentration of 0.5 mg/mL shows the peak of this cytostatic drug. The standard calibration curve of cisplatin, namely, the dependence of the peak area on the chromatogram on the drug concentration, was constructed to assess the diffusion of cisplatin from the hydrogels that differ in type of pectin (citrus or apple) and the concentration of polymers. The chromatogram of the aqueous medium taken 45 min after the start of diffusion of cisplatin from the hydrogel is shown in Figure 13. The retention time of cisplatin on the chromatogram (peak 3) exactly coincides with the retention time of the standard sample of this drug (2.5 min). Peaks 1 and 2 belong to chitosan hydrochloride and pectin, respectively. HPLC studies have shown that a small amount of both polysaccharides, probably not bound into a polymer network, is released at a low rate in the first hours of the experiment, then this process stops.

**Figure 14:**
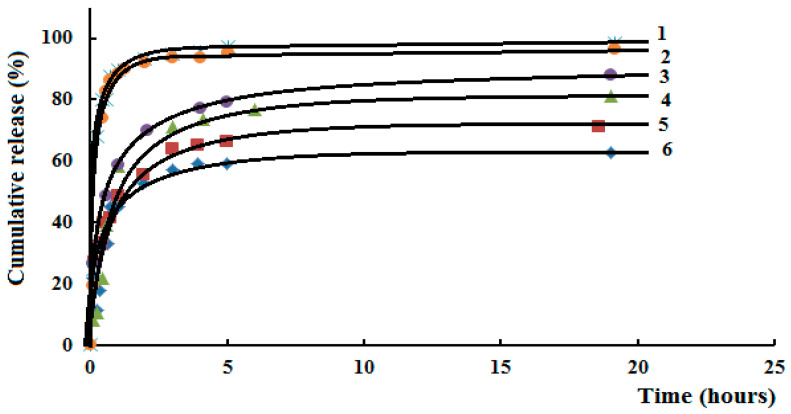
Kinetic curves of cisplatin release from film (1, 2) or hydrogel (3–6) based on chitosan hydrochloride and apple (1, 3, 5) or citrus (2, 4, 6) pectin in saline solution. Concentration of polymers, mg/mL of hydrogel: 6.4 (1, 2, 5, 6) or 3.2 (3, 4). Data are expressed as mean ± SD; *n* = 3.

Studies have shown that the use of hydrogels as carriers, in contrast to films of a similar composition, makes it possible to reduce the drug release rate, which is associated with a more developed surface of the three-dimensional structure. In the first minutes of the experiments, a burst release of cisplatin is observed from the polymer matrix; then, after 1–2 h, the release rate decreases and the cumulative release gradually reaches a plateau (Figure 14). The greater the concentration of polymers in the hydrogel, the slower the drug is released, which is associated with a denser crosslinked network due to various polymer interactions. The use of citrus pectin instead of apple pectin also makes it possible to somewhat reduce the release rate, which is apparently associated with more elastic rheological properties of hydrogels based on higher molecular mass pectin.

## 3. Conclusions

Physically crosslinked hydrogels were successfully prepared by mixing solutions of pectin and chitosan under mild conditions at room temperature without isolating a dispersion medium; the gelation time was about 1 min. The crosslinking of the polymers occurs due to the formation of a polyelectrolyte complex between pectin and chitosan. It was established that the viscosity of citrus pectin hydrogel is about four times higher than that of apple pectin hydrogel, which is likely associated with differences in their molecular weights. Studies of the swelling kinetics and stability of hydrogels have shown that their properties are affected by the presence of inorganic ions in the composition of both the hydrogel and the environment. In this case, the higher the concentration of polymers in the gel, the greater its swelling. The smallest weight loss of the hydrogel is observed in a medium similar in composition to its dispersion medium (neutral pH). It has been shown that in the presence of hyaluronidase, the hydrogel undergoes degradation. It has been shown that cisplatin does not prevent the formation of a hydrogel based on chitosan and pectin. The prolonged release of cisplatin was shown to be dependent on the polymers concentrations by using high-performance liquid chromatography.

## 4. Materials and Methods

### 4.1. Materials

Chitosan hydrochloride (50 kDa) was purchased from «Bioprogress», Losino-Petrovsky, Russia, low molecular weight apple pectin (30 kDa) and high molecular weight citrus pectin (70 kDa) were obtained from «Herbstreith & Fox KG», Neuenbürg, Germany. Hyaluronidase was purchased from NPO «Microgen», Moscow, Russia. Cisplatin was obtained from FGBU «NMIC oncology N.N. Blokhin», Moscow, Russia. Inorganic salts NaCl, CaCl_2_, AlCl_3_, Na_2_HPO_4_, NaH_2_PO_4_ were purchased from Sigma Aldrich, Schnelldorf, Germany. All polymers and reagents were of analytical grade.

### 4.2. Preparation of Hydrogels

To dissolve the polysaccharides, we used distilled water, saline in the absence or presence of cisplatin at a concentration of 1.0 mg/mL, or phosphate buffer (pH = 7.4). Solutions of chitosan hydrochloride and pectin were prepared separately at concentrations of 4–8% and 1–2%, respectively, and were then mixed in equivalent amounts by shaking the vial. The formation of the hydrogel was judged by the loss of fluidity of the system over time when the vial was inverted.

Hydrogel structures were characterized using FT-IR spectroscopy with a Tensor-27 BRUKER, Germany. The degree of deacetylation of chitosan and the degree of esterification of pectins were determined using acid–base titration with potentiometric determination of equivalence points.

### 4.3. Rheological Studies of Hydrogels and Polymer Solutions

The rheological parameters of the hydrogels were measured under shear deformation conditions using a Haake Viscotester iQ rotational viscometer, Thermo Fisher Scientific, Karlsruhe, Germany, with a system of coaxial cylinders (radius of the outer cylinder r_o_ = 8.5 mm; ratio of the radii of the outer and inner cylinders r_o_/r_i_ = 1.1). The effective viscosity was measured in the shear rate range from 0.1 to 300.0 s^−1^.

To establish the yield strength of hydrogels, the obtained flow curves in the nonlinear region were approximated by the Herschel–Bulkley rheological model [32], which describes the flow of viscoplastic materials:

(1) $$\tau =\tau_{0}+K\dot{\gamma^{n}},$$

where *τ*_0_—Herschel–Bulkley yield strength (Pa), *K*—consistency factor, $\dot{\gamma }$—shear rate (s^–1^), *n*—current index.

### 4.4. Swelling Measurements

The preliminarily weighed dry gel 0.1 g was placed in 5 mL of distilled water or a salt solution with a concentration of 0.5–2.0%, and the change in its mass over time was monitored by the gravimetric method. The degree of swelling was calculated using the following equation:

(2) $$Swelling\;ratio\;\left(\%\right)=\frac{m-m_{0}}{m_{0}}\times 100\%,$$

where *m*—mass of the hydrogel at the moment *t*, *m*_0_—initial mass of the hydrogel. 

### 4.5. Stability Measurements

Nonenzymatic degradation of the hydrogel over time was assessed using a stability test. After the gel formation, the samples were hydrated in solution and incubated at 37 °C, controlling the weight at different time points. The percentage of weight loss (*W_L_*) was calculated using the following Equation (2):

(3) $$Weight\;loss\;\left(\%\right)=\left(1-\frac{W_{0}-W_{i}}{W_{0}}\right)\times 100\%,$$

where *W*_0_ is the initial weight of the hydrogel at *t* = 0 after thermal gelation, and *W_i_* is the weight of the hydrogel at different times.

### 4.6. Enzymatic Degradation

The degradation of the hydrogel was studied by placing a weighed sample (1.0 g) into a flask with 5 mL of saline solution, containing the hyaluronidase enzyme at a concentration of up to 64 U/mL at 20 or 40 °C. At specified time intervals, hydrogel was removed from the saline solution, lyophilized, and weighed. The weight loss of the hydrogel was calculated using the following equation:

(4) $$Gel\;degradation\;\left(\%\right)=\left(1-\frac{W_{0}-W_{t}}{W_{0}}\right)\times 100\%,$$

where *W*_0_ weight of initial hydrogel, *W_t_*—weight of hydrogel at the moment *t.*

### 4.7. In Vitro Cisplatin Release

The release of cisplatin from hydrogels differing in the total concentration of polymers and the nature of pectin and films was evaluated using high-performance liquid chromatography. Samples containing 1 mg/mL cisplatin were incubated in saline at room temperature. After 5 min, 15 min, 30 min, and then every hour samples of the solution were taken and analyzed using HPLC. After processing the obtained data, using the calibration curve of cisplatin, we obtained the dependence of the release of the drug from the polymer matrix into the solution over time.

Quantitative analysis of cisplatin diffusion was carried out on a Shimadzu LC-20 liquid chromatograph with a spectrophotometric diode array detector, Shimadzu Corporation, Kyoto, Japan. A column with a Pursuit XRs C18 phase 250 × 4.6 mm, 5 μm was used, and the eluent of acetonitrile composition: water = 20:80 (vol. %) was used as the mobile phase. The flow rate was 1 mL/min. Detection was carried out at a wavelength of 215 nm.

All the experiments were conducted three times for each sample.

## Data Availability

Not applicable.
